# Clinical and Radiographic Characteristics of Pulmonary Nocardiosis: Clues to Earlier Diagnosis

**DOI:** 10.1371/journal.pone.0090724

**Published:** 2014-03-03

**Authors:** Junjun Chen, Hua Zhou, Panfeng Xu, Pei Zhang, Shanni Ma, Jianying Zhou

**Affiliations:** Department of Respiratory Diseases, The First Affiliated Hospital, College of Medicine, Zhejiang University, Hangzhou, Zhejiang, People’s Republic of China; Fundacion Huesped, Argentina

## Abstract

**Background and objectives:**

Pulmonary nocardiosis (PN) is a rare but life-threatening disease that is caused by *Nocardia* spp. The aim of this study was to characterize the common risk factors, clinical features, imaging findings, treatment and outcomes of PN, which are useful for an early diagnosis and patient management.

**Methods:**

From January 2009 to June 2013, a retrospective study was performed on all PN cases that were diagnosed at our hospital.

**Results:**

The study included 17 patients who were diagnosed with PN. Of these patients, 4 developed concomitant disseminated disease. A male predominance was observed among the patients with PN (76.47%). The most common risk factors were corticosteroid therapy (64.71%), diabetes mellitus (29.41) and chronic lung disease (23.53%). Cough and fever were the most common symptoms (94% and 71%, respectively). One or more nodules or masses (82.35%) and consolidations (58.82%) were the most frequent radiologic abnormalities, and cavitation mostly occurred within two weeks. The median time to diagnosis was 25 days. Overall, the mortality rate was 18.75% for PN, and death was most frequent among patients who received immunosuppressive drugs. For the patients with central nervous system involvement, the mortality rate was 50%.

**Conclusion:**

PN remains a rare opportunistic infection that mainly affects immunocompromised patients. A high clinical index of suspicion is necessary for an early diagnosis and timely treatment in immunocompromised patients who present with new nodules or masses evolving into cavitation in a short amount of time.

## Introduction

*Nocardia*, which was first described in 1888 by Edmund Nocard [1], is a genus of aerobic Gram-positive modified acid-fast stain-positive bacteria that are not part of the normal human flora and are not a common laboratory contaminant [2]. The organisms are widely distributed in dust, soil, water and vegetable matter; however, they may become airborne in dust particles and lead to infection via inhalation. Nocardiosis is an opportunistic infection that mainly affects immunocompromised patients, such as patients with acquired immunodeficiency syndrome (AIDS), patients with long-term steroid use or transplant recipients. Nocardiosis can also cause life-threatening disease in immunocompetent patients [3]–[5].

An important trait of this organism is its ability to disseminate to most organs, and pulmonary involvement is most commonly encountered [6]. Although the incidence of pulmonary nocardiosis (PN) is low, the mortality rate of PN is high when this disease is not diagnosed in a timely manner [5], [7]. Furthermore, a significant amount of time is needed to establish a diagnosis of nocardiosis, which has been reported to be as long as 6 weeks [8]. Therefore, patients would benefit from a rapid diagnosis using clinical and radiological findings. Nevertheless, most of our knowledge about PN in China is based on case reports, and case series are needed to provide more information on this disease. This study aimed to retrospectively analyze the common risk factors, clinical features, imaging findings, treatment and outcomes of PN patients because this information will be useful for an early diagnosis and patient management.

## Materials and Methods

This study was a retrospective analysis that was conducted at the First Affiliated Hospital of Zhejiang University. Patients with a diagnosis of PN from January 2009 to June 2013 were included in the study. Patients with extrapulmonary nocardial infection but no evidence of pulmonary disease were excluded. The medical records of all the patients who were included in this study were analyzed to obtain demographic data, host immune status, symptoms, radiographic presentation, treatment, follow-up and outcomes.

### Ethics Statement

The study was approved by the Ethics Committee of The First Affiliated Hospital, Zhejiang University, School of Medicine. All the patients provided written informed consent and understood that their hospital data would be used for research.

### Definitions

The diagnosis of PN required the isolation of *Nocardia* species from respiratory samples, including sputum, bronchoalveolar lavage and fluid obtained from lung punctures. In addition, the presence of pertinent clinical symptoms and signs was required for a PN diagnosis. Disseminated nocardiosis was diagnosed when infection was present in two non-contiguous organs or in the central nervous system (CNS). Patients with clinically diagnosed chronic obstructive pulmonary disease (COPD), bronchiectasis or allergic bronchopulmonary aspergillosis were considered to have chronic lung disease. Long-term glucocorticoid usage was defined as taking at least 10 mg of prednisolone (or prednisolone equivalent) per day for more than 1 month before the development of nocardiosis.

### Imaging Techniques and Analysis

Computed tomography (CT) was performed using 16-slice or 64-slice systems (Toshiba Aquilion 16 CT Scanner; Brilliance iCT and 64-channel systems). The protocol was as follows: end-inspiratory acquisition, 120 kV, 200–250 mAs and a thickness of 5–6.5 mm. Intravenous contrast was used in all of the patients. Two experienced radiologists evaluated the CT images, and consensus was achieved by negotiation. The observers assessed the scans for the presence of nodules, masses, ground-glass opacities (GGOs), consolidations, cavitation, pleural effusions and air bronchograms. The criteria for these findings are defined in the Glossary of Terms by the Fleischner Society [9].

### Microbiological Analysis

The identification of *Nocardia* spp. was based on the presence of aerobic growth, colonial and microscopic morphology, positive Gram staining and positive modified acid-fast staining. The cultures were incubated for 2–7 days at 35–36°C on blood agar and chocolate agar [4], [10]. Molecular techniques were not routinely performed.

## Results

### Patient Characteristics

During the study period (January 2009 to June 2013), we identified 22 patients with positive *Nocardia* cultures from various sites in the body. Overall, five patients were excluded due to the isolation of *Nocardia* from blood (2 patients) and drainage (3 patients) with no evidence of pulmonary disease. Therefore, the data from 17 patients (4 female and 13 male patients) were included in the study. Of these patients, four developed concomitant disseminated disease with CNS involvement (2 patients), cutaneous and pericardial abscesses (1 patient) and cutaneous and pelvic cavity abscesses (1 patient). Notably, one patient who underwent renal transplantation was included twice due to relapsed infection with *Nocardia* spp. Of the 17 patients with PN, 11 were immunocompromised for various reasons, four had chronic lung disease, and two had no predisposing factors. Furthermore, the leading common immunosuppressive therapy in patients with PN was corticosteroids (11 patients). The demographic features of the patients, their underlying diseases and the immunosuppressive therapies are described in Table 1.

**Table 1:** Demographics, underlying disease and immunosuppressive therapies in 17 patients with PN.

	No.	(%)
Mean age, years	51.1±9.5	
Sex distribution		
Male	13	76.47
Female	4	23.53
Underlying disease		
Solid tumor^a^	2	11.76
Human immunodeficiency virus infection	1	5.88
Solid-organ transplantation^b^	3	17.65
Chronic lung disease	4	23.53
Autoimmune disorders^c^	3	17.65
Chronic renal failure	3	17.65
No underlying disease	2	11.76
Chronic drug use		
Chemotherapy	1	5.88
Long-term glucocorticoid usage	11	64.71
Immunosuppressants	3	17.65

a Primary lung cancer (1), mediastinal neuroendocrine tumor (1) and primary liver cancer (1).

b Liver transplantation (1) and renal transplantation (2).

c Systemic lupus erythematosus (1), uveitis (1) and polymyositis (1).

### Clinical Features

**Figure 1:**
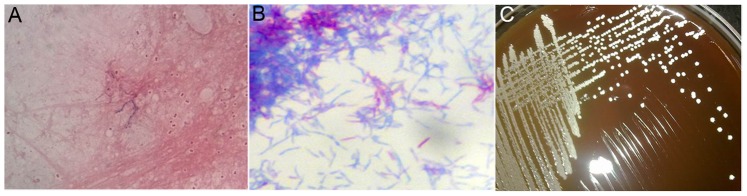
A 45-year-old woman on corticosteroid therapy for systemic lupus erythematosus and lupus nephritis was infected with *Nocardia* spp. A, Gram staining demonstrates a delicate, beaded, branching filamentous bacillus; B, Modified acid-fast staining was weakly positive for *Nocardia*; C, Rugose colonies of *Nocardia* on blood agar plates.

Cough was the most common presenting symptom, occurring in 16 patients (94.12%), followed by fever (n = 12, 70.59%), dyspnea (n = 8), fatigue (n = 5), chest pain (n = 5), neurological signs (n = 3), cellulitis (n = 2) and diarrhea (n = 2). Hemoptysis was observed in one patient who had bronchiectasis. Of 17 patients, seven were initially misdiagnosed (aspergillosis [n = 5], cryptogenic organizing pneumonia [n = 1] and pulmonary vasculitis [n = 1]). Invasive diagnostic procedures (fine-needle biopsy) were performed in five patients and were successfully used to diagnose PN. In the remaining 12 patients, *Nocardia* spp. was isolated from sputum culture (Fig. 1). In four patients with a positive sputum culture, *Nocardia* spp. was isolated in other samples (abscess puncture in two patients, pericardial effusion in one patient and blood culture in three patients). Pulmonary coinfection was diagnosed in three patients (*aspergillus* in 2 patients and *Klebsiella pneumoniae* in 1 patient).

The complete blood cell counts in the 17 patients revealed that seven (41.18%) had leukocytosis and 13 (76.47%) had neutrophilia. An increase in inflammatory markers (C-reactive protein, CRP; erythrocyte sedimentation rate, ESR) was observed in 81.25% (13/16) and 93.33% (14/15) of the patients, respectively. Species identification was available in only two patients (*Nocardia asteroides*).

The course of disease was subacute in most patients. The mean time from the onset of symptoms to diagnosis was 29.5±22.5 days (median 25 days). The mean duration from admission to diagnosis was 18.0±13.3 days (median 12 days). Table 2 shows the clinical characteristics of the patients.

**Table 2:** The clinical characteristics of 17 patients with PN.

Clinical characteristics	No.	(%)
Cough	16	94.12
Fever	12	70.59
Dyspnea	8	47.06
Fatigue	5	29.41
Chest pain	5	29.41
Cellulitis/cutaneous abscess	2	11.76
Neurological signs	3	17.65
Diarrhea	2	11.76
Hemoptysis	1	5.88
Site of culture		
Sputum	12	70.59
Puncture fluid of lung or lung tissue	5	29.41
Abscess puncture	2	11.76
Pericardial effusion	1	5.88
Blood	3	17.65
Laboratory examination		
Leukocyte count >10,000	7	41.18
Neutrophilia (>80%)	13	76.47
ESR>20 mm/h	14	93.33
CRP>8 mg/dl	13	81.25
Duration from admission to diagnosis(days)	18.0±13.3	

### Radiographic Findings

All 17 patients received CT scans. The pulmonary abnormalities that were detected on the initial CT scans are summarized in Table 3. The most common radiologic findings were one or more nodules or masses, which occurred in more than 80% of the patients. No significant differences were found in the incidence of CT abnormalities between the male and female patients.

**Table 3:** Radiologic characteristics of 17 patients with PN.

Major radiographic findings	No. of patients	%
Nodules	14	82.35
Cavitation	13	76.47
Pleural effusion	11	64.71
Consolidation	10	58.82
Ground-glass opacity	10	58.82
Air bronchogram	8	47.06
Masses	7	41.18
Bronchiectasis	5	29.41
Lymphadenopathy	4	23.53
Pericardial fluid	4	23.53

**Figure 2:**
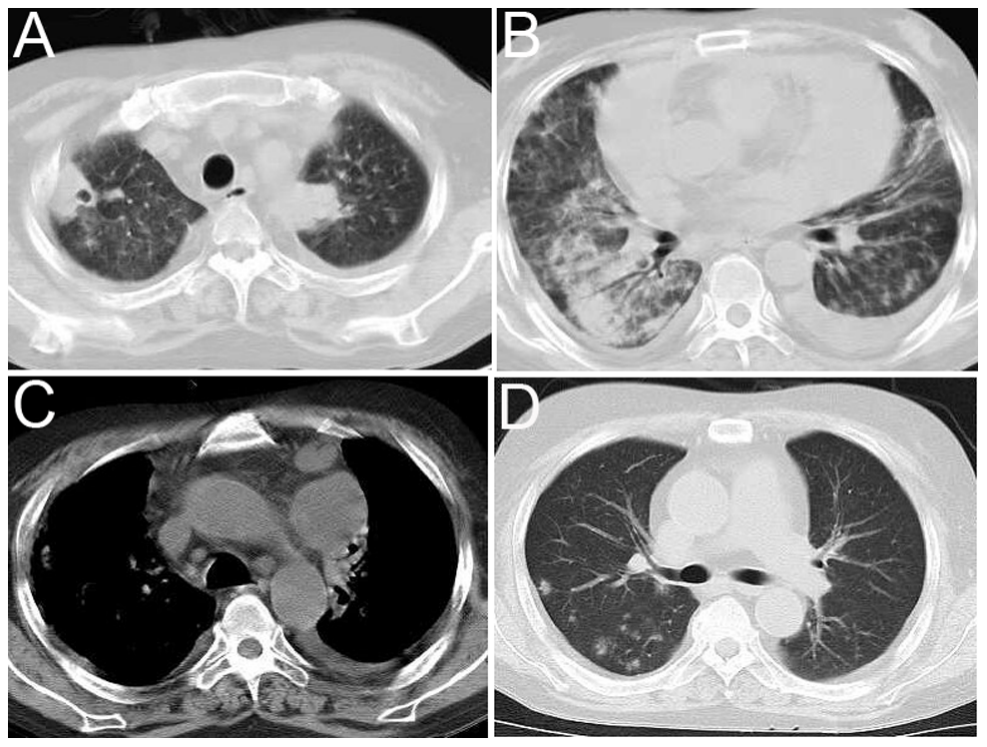
A 45-year-old man on immunosuppressive therapy after renal transplantation was infected with *Nocardia asteroides*. A–C, Axial CT images reveal multiple patterns of disease and lymphadenopathy; D, An axial CT image reveals small pulmonary nodules with relapsed infection two years after the initial diagnosis.

**Figure 3:**
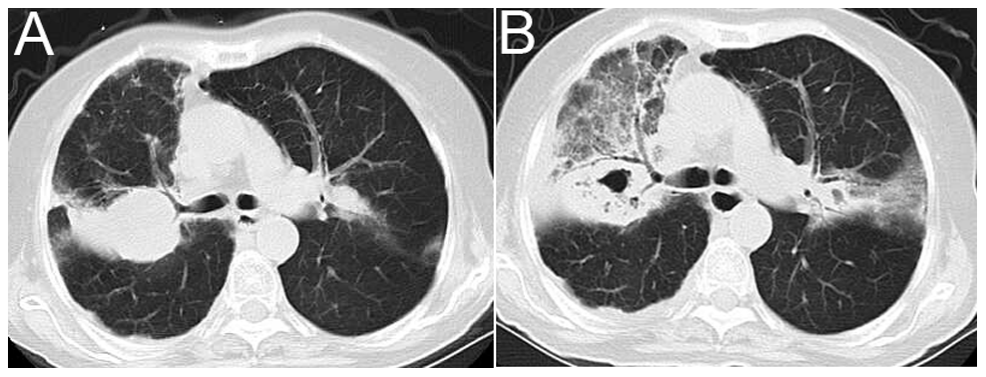
A 63-year-old woman with COPD and *Nocardia* spp. infection. A, An axial CT image reveals a lobar consolidation in the right upper lobar; B, Axial CT imaging was performed 12 days later and the images reveal a cavitary consolidation on the right and GGO changes. Note the prominent air bronchogram.

**Figure 4:**
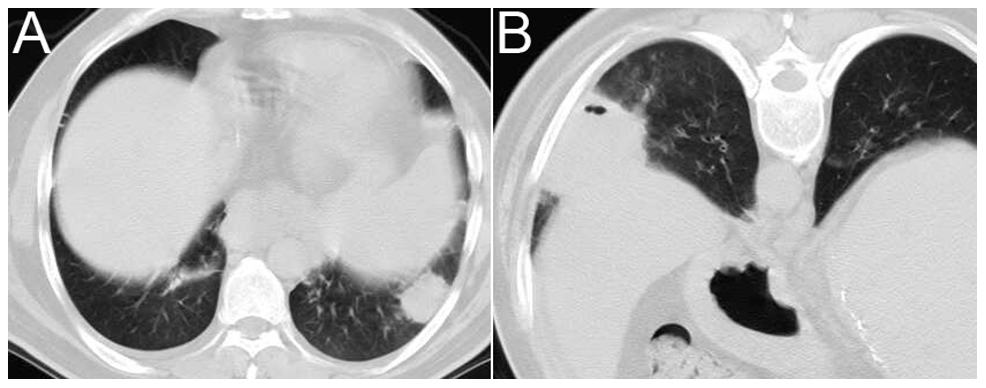
A 42-year-old man after liver transplantation. He suffered from fever 15 days after the liver transplant. Thoracic CT shows a solidary nodule in the left lower lobe (A). CT-guided puncture of the left lower lobe was performed 20 days after the liver transplant (B), and a punctured tissue culture revealed *Nocardia* spp. infection.

Nodules (Fig. 2A) were observed in 14 (82.35%) patients, with seven presenting masses concurrently. These lesions were solitary in one patient but multiple in the other 13 patients. Most of the nodules and masses had poorly defined margins, and a peripheral predominance was observed in seven patients. Nodules with cavitation (Fig. 2A) were detected in 12 patients, which were eccentric with irregular and thickened walls. Furthermore, cavitation occurred within two weeks in the majority of these patients (66.67%, 8/12) (Fig. 3,4).

Consolidations (Fig. 2B) were observed in 10 (58.82%) patients, and cavitation was detected in three of these patients. Air bronchograms were found in eight of these patients, whereas GGOs were observed in seven of these patients (Fig. 3B). Multiple nodules were observed in seven patients with consolidations, which were coupled with masses in three cases.

Pleural effusions (Fig. 2B–C) were a frequent finding, which were detected in 11 cases, including six cases with bilateral effusions, three with right unilateral effusions and two with left unilateral effusions. Other associated findings included lymphadenopathy (n = 4, 23.53%), bronchiectasis (n = 5, 29.41%), areas of GGO (n = 10, 58.82%) and pericardial fluid (n = 4, 23.53%).

### Treatment and Outcomes

After a diagnosis of PN was established, one patient was immediately referred to another medical institution for continuous hemoptysis and was lost to follow-up. The antibiotic treatment regimens that were administered to the patients are listed in Table 4. Four patients received monotherapy as a first-line treatment (three patients were treated with co-trimoxazole and one patient was treated with meropenem). However, this therapy was changed to combination therapy in one patient. The remaining patients initially received combination therapy. Adverse events occurred in two patients during treatment, which resulted in a change of the treatment regimen. Myelosuppression occurred in one patient on linezolid therapy, and severe nausea occurred in one patient on co-trimoxazole therapy. Surgical intervention was necessary in one patient with empyema who received co-trimoxazole plus meropenem.

**Table 4:** Specific antibiotic therapy and mortality.

Treatment approaches	n treated patients/n deaths
Co-trimoxazole	3/0
Co-trimoxazole + carbapenem	6/2
Co-trimoxazole + linezolid	2/0
Carbapenem	1/0
Carbapenem + minocycline	1/0
Triple therapy^a^	3/1

a Co-trimoxazole+amikacin+linezolid (1), Imipenem+co-trimoxazole+amikacin (1), co-trimoxazole+ceftriaxone+chloromycetin (1).

Long-term follow-up was available for 16 patients, of whom 13 were cured and three died (18.75%). All the deaths occurred in immunocompromised patients. The mortality rate was higher among patients with disseminated disease (25% vs. 16.7%), CNS involvement (50% vs. 14.3%), immunosuppressive drug use (27.3% vs. 0%) and human immunodeficiency virus (HIV) infection (100%) than in the other patients in this cohort.

## Discussion

Nocardiosis is an opportunistic infection that mainly affects immunocompromised hosts, especially individuals with altered cellular immunity; however, this infection can occur in immunocompetent persons as previously reported [3]–[5]. In this study, the major underlying condition in the PN patients was prolonged corticosteroid therapy, which was present in more than 60% of our patients. Another important underlying condition in our cohort of patients was chronic lung disease (23.53%). COPD was the most common respiratory disease in patients with PN. COPD patients typically receive prolonged corticosteroid treatment; however, Riviere et al. suggested that COPD may be the only risk factor for nocardiosis [11]. In patients with COPD, bacterial colonization of the bronchus alters ciliary motility and causes epithelial damage, which facilitates the growth of *Nocardia* [8]. In addition, malignancies, solid-organ transplantation, HIV infection, diabetes mellitus and autoimmune diseases were determined to be risk factors in our patient population, which is consistent with the results of other studies [8], [12].

Previous studies [6], [13] have found a male predominance among patients with *Nocardia* infection, which was demonstrated in this study (76%) and may be ascribed to hormonal effects on the virulence or growth of *Nocardia* [14].

The disease onset was sub-acute in most patients (58.82%). Similar to previous reports, the most frequent symptoms in our patients included cough and fever (94% and 71%, respectively). Additionally, shortness of breath, fatigue, chest pain, cellulitis and neurological signs were found in our series and in previous reports [8], [12], [13].

The manifestations of PN on chest CT can be diverse. In this study, the most common CT manifestations were single or multiple nodules (82.35%) and airspace consolidations (58.82%). The high frequency of these lesions is consistent with findings from other studies [15], [16]. Notably, cavitation coupled with nodules, masses or consolidations was observed in most patients (76.47%), and this rate of cavitation is higher than previous rates, which ranged from 18–60% [15]–[17]. This result may be due to various predisposing factors in our patient population or to the higher scanner resolution that was used in this study and in other recent studies. Furthermore, more than 60% of these cavitations occurred within 2 weeks. Other findings included GGOs, air bronchograms, bronchiectasis, lymphadenopathy, pleural effusion and pericardial fluid, which have been observed in other studies [15]–[19].

A diagnosis of PN is often delayed, and the median time interval between symptom onset and diagnosis was 25 days (6–84 days) in our series. This delay may be attributed to the non-specific clinical presentations of PN and the initial laboratory findings in patients with PN, which mimic pulmonary tuberculosis, invasive fungal disease and lung cancer. A diagnosis of PN using radiologic findings may be difficult because this method is often associated with poor specificity; however, correlating disease patterns and progression with clinical history may provide crucial diagnostic clues. Therefore, nocardiosis should be considered in the differential diagnosis of immunocompromised patients who present with new nodules or masses, and suffered from pneumonia with poor response to treatment, particular when these manifestations are associated with brain and extrapulmonary abscesses.

A diagnosis of *Nocardia* infection requires the isolation and identification of organisms from a clinical specimen. The most effective sample for a diagnosis of PN is sputum that is obtained by non-invasive methods. Nevertheless, when patients cannot expectorate, invasive methods are reasonable and recommended to obtain a rapid diagnosis, and these methods include bronchoalveolar lavage and percutaneous lung puncture biopsy. The data from our series indicated that 29% of the patients were diagnosed according to the results of invasive methods and that all of the patients were successfully treated.

No randomized prospective clinical trials have been performed to determine the most appropriate therapeutic agent, administration route and treatment duration for patients with *Nocardia* infection. For many years, co-trimoxazole has been the first-line drug of choice for the treatment of nocardiosis because most studies have demonstrated that *Nocardia* is highly susceptible to this drug [12], [13]. However, a recent study detected a 42% rate of resistance to co-trimoxazole among 765 isolates of *Nocardia* [20]. Moreover, different species have different antimicrobial resistance profiles. Therefore, initial combination therapy with two or more active agents, including co-trimoxazole, amikacin, cephalosporin, carbapenem, minocycline and linezolid, is recommended for patients with disseminated or severe nocardiosis [6].

The mortality rate of the patients with PN was 18.75% in this study, which is similar to findings in other studies [8], [12], [13]. In the analysis of predisposing factors and sites of infection, the mortality rates were 27.3% (3/11) in immunosuppressed patients and 50% in patients with CNS involvement, and no mortality was observed in patients with disease that had disseminated to the skin and soft tissues.

## Conclusion

PN remains a rare infection. However, patients who are at a high risk of PN include patients on corticosteroid therapy, patients who undergo post-organ transplantation, patients with chronic lung disease and HIV-positive patients. In this study, the most common symptoms were cough and fever, which were occasionally combined with extra-pulmonary symptoms and signs. The CT presentation of PN at the time of diagnosis was heterogeneous. Nodules, masses and consolidations were the most common presentations, which progressed to cavitation in a short amount of time. Therefore, PN must be suspected in patients with these risk factors and imaging findings. Moreover, initial combination therapy with two or more active agents is recommended for patients with disseminated or severe nocardiosis.
